# Concurrent Osteosarcoma Theranostic Strategy Using Contrast-Enhanced Ultrasound and Drug-Loaded Bubbles

**DOI:** 10.3390/pharmaceutics11050223

**Published:** 2019-05-08

**Authors:** Tai-Tzung Kuo, Chung-Hsin Wang, Jir-You Wang, Hong-Jen Chiou, Ching-Hsiang Fan, Chih-Kuang Yeh

**Affiliations:** 1 Department of Biomedical Engineering and Environmental Sciences, National Tsing Hua University, Hsinchu 30013, Taiwan; 4348@mmh.org.tw (T.-T.K.); chwang@trust-biosonics.com (C.H.W.); s1024@msn.com (C.-H.F.); 2 Department of Neurosurgery, Hsin-chu Mackay Memorial Hospital, Hsinchu 30071, Taiwan; 3 Department of Orthopaedics and Traumatology, Taipei Veterans General Hospital, Taipei 11217, Taiwan; jywang7@vghtpe.gov.tw; 4 Department of Orthopaedics, Therapeutical and Research Center of Musculoskeletal Tumor, Taipei Veterans General Hospital, Taipei 11217, Taiwan; 5 Institute of Traditional Medicine. National Yang-Ming University, Taipei 11221, Taiwan; 6 Department of Radiology, Taipei-Veterans General Hospital, Taipei 11217, Taiwan; hjchiou@vghtpe.gov.tw; 7 Institute of Nuclear Engineering and Science, National Tsing Hua University, Hsinchu 30013, Taiwan

**Keywords:** microbubbles, contrast-enhanced ultrasound, osteosarcoma, enhanced permeability, drug-loaded, ultrasound contrast agents, therasostics

## Abstract

Osteosarcoma (OS) is the most common bone tumor in children and teenagers. The multidrug resistant property of OS produces a major obstacle to chemotherapy, since the effective drug dose cannot be achieved via conventional drug delivery routes without serious systemic cytotoxicity. Microbubbles in conjunction with ultrasound (US) has recently been shown to spatially and temporally permeabilize the cellular membrane, promoting drug penetration into tumors. Here, we investigated whether drug (doxorubicin, DOX)-loaded bubbles (DOX-bubbles) can serve as drug-loaded carriers in combination with US in order to facilitate tumor drug delivery. The proposed bubbles have a high payload capacity (efficiency of 69.4 ± 9.1%, payload of 1.4 mg/mL) for DOX. In vitro data revealed that when used in combination with US (1-MHz), these DOX-bubbles facilitate DOX entering into tumor cells. In tumor-bearing animals, DOX-bubbles + US could provide 3.7-fold suppression of tumor growth compared with the group without insonation (1.8 ± 0.9 cm^3^ vs. 8.5 ± 2.2 cm^3^) because of the acceleration of DOX-induced tumor necrosis. In the meantime, the tumor perfusion and volume can be monitored by DOX-bubbles with contrast-enhanced ultrasound imaging. Our data provide useful information in support of translating the use of theranostic US-responsive bubbles for regulated tumor drug delivery into clinical use.

## 1. Introduction

Osteosarcoma (OS) is the most common kind of bone cancer in children and teenagers [1]. It is characterized by high proclivity for early systemic metastases and local invasion [2]. The current approved clinical treatments for OS include surgery and chemotherapy (such as doxorubicin, DOX) [3,4]. The cure rate in OS patients ranges from 15 to 20% with surgery alone, but improves to nearly 70% when the surgery is performed in conjunction with chemotherapy [4]. Despite advances in surgical techniques and neoadjuvant chemotherapy regimens, the failure of cure with OS is still about 30%, which is mainly due to the development of multidrug resistance [4,5]. Developing novel chemotherapeutic strategies to overcome multidrug resistance of OS is necessary.

The decrease in intracellular drug efflux caused by P-glycoprotein (P-gp) plays an important role in multidrug resistance in OS therapy. The limited drug accumulation results in failure to respond to DOX [6]. In order to overcome the drug resistance caused by P-gp, numerous approaches have been broadly proposed, including the administration of DOX-carrying lipid-based nanoparticles or combinations of DOX with other chemotherapeutic agents (i.e., cisplatin, methotrexate) [7,8]. However, a nanoscale drug delivery system probably affects all organs during circulation, leading to accumulation of the cytotoxic agents in undesired areas; several classes of administrated drug for treating OS can lead to systemic cytotoxic effects.

The use of ultrasound (US) exposure in the presence of microbubbles has been approved in pre-clinical studies as a noninvasive and reversible approach to enhance permeability in the local area, leading to a transient opportunity for facilitating delivery of therapeutic substances to a target site [9,10,11,12,13]. Further, this approach can produce cardio- and nephrotoxicity due to direct exposure to the chemo-drug in blood circulation [14]. The encapsulation of chemo-drug can protect normal tissues from toxicity and direct exposure. 

**Figure 1:**
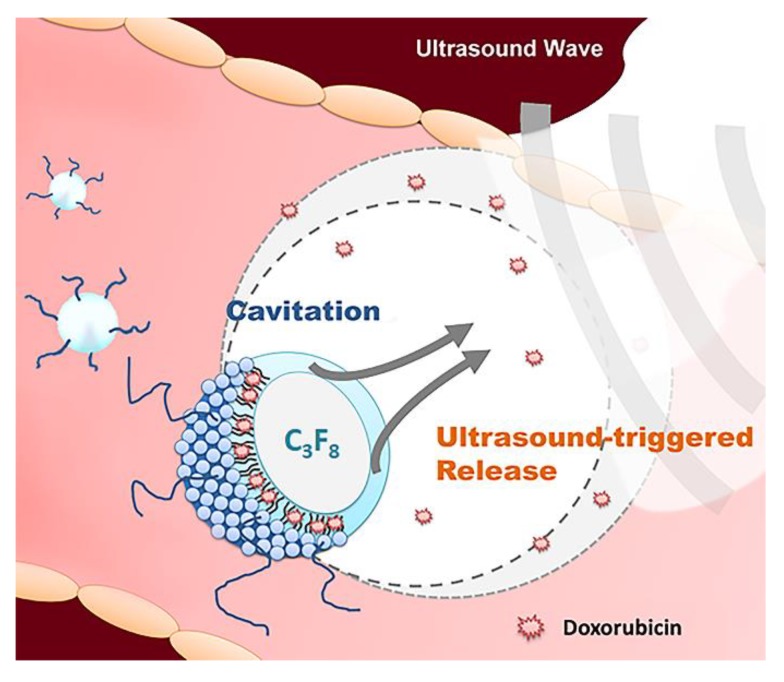
Diagram showing drug-loaded bubbles and ultrasound exposure to actively trigger drug release for osteosarcoma theranosis.

In numerous pilot studies, microbubbles have been identified as a mechanism for encapsulating drugs, such as DOX and paclitaxel, for drug delivery [14,15,16,17]. The carried drug within bubbles could be triggered release upon US sonication, achieving local drug delivery while decreasing the drug exposure in blood circulation [18,19,20,21,22]. Additionally, bubbles exhibit a bi-functional characteristic that merges ultrasonic diagnosis with therapy. In this study, we aimed to build a DOX-loaded bubble (DOX-bubbles) system that provided: (1) a high drug (DOX) payload carrier with high ultrasonic sensitivity, (2) enhanced drug delivery triggered by US sonication, (3) ultrasonic imaging to assess tumor microperfusion information. This concurrent osteosarcoma theranostic strategy is shown in Figure 1. The fabrication and characteristics of DOX-bubbles, and their ability to improve cellular drug delivery with US is described herein. The feasibility of utilizing DOX-bubbles with contrast-enhanced ultrasound imaging (CEUS) for evaluating perfusion information of tumor was also assessed. Finally, the cancer treatment outcome with our proposed strategy was estimated using an animal OS tumor model, demonstrating the theranostic potential.

## 2. Material and Methods

### 2.1. Materials

The pure lipid-based and perfluoropropane (C_3_F_8_)-filled bubbles agent, was obtained in cooperation with Trust Bio-sonics, Inc. (Deliver, Hsinchu, Taiwan). The lipid shell consists primarily of 1,2-distearoyl-sn-glycero-3-phosphocholine (DSPC, Avanti Polar Lipids, Alabaster, AL, USA), 1,2-dioctadecanoyl-sn-glycero-3-phospho-(10-rac-glycerol) (DSPG, Avanti Polar Lipids, Alabaster, AL, USA), and 1,2-distearoyl-sn-glycero-3-phosphoethanolamine-*N*-[methoxy(poly(ethyleneglycol))-2000] (DSPE-PEG2000, Avanti Polar Lipids, Alabaster, AL, USA). The phosphate-buffered saline (PBS), glycerol, and doxorubicin (DOX) were purchased from Sigma-Aldrich (St. Louis, MO, USA).

### 2.2. Preparation of Doxorubicin-Loaded Bubbles

The molar ratio of DSPC, DSPG and DSPE-PEG2000 were 21:21:1. This information has been added in the revised manuscript (line). To load the bubbles with DOX molecules, a DOX aqua solution (with deionized water, in the concentration of 10 mg/mL) was injected into pure bubbles without drug loading agent (Deliver) to mix with the formulated lipids [14,16]. The mixing ratio was based on the recommendation of 100 μL DOX solution mixed with 400 μL lipid solution. The final DOX concentration of the lipid solution was 2 mg/mL. To further incorporate the DOX molecules into the lipid membranes, the mixed solution was placed in a 60 °C water bath for 30 min. The mixed solution was then cooled to room temperature and agitated using an amalgamator (AM-1, MONITEX Industrial Co., New Taipei City, Taiwan) for 60 s to produce the DOX-loaded bubbles. The sample was centrifuged at 1000g for 3 min to isolate DOX-bubbles from free drug molecules. After centrifugation, the solution layer was removed and then re-filled with fresh PBS to re-suspend the bubbles. The pure bubbles without drug loading and commercially available microbubbles (SonoVue^TM^, Bracco Diagnostics Inc., Milan, Italy) were used for comparison.

### 2.3. Characterization of DOX-Loaded Bubbles

The size distribution and concentration of DOX-bubbles, pure bubbles, and SonoVue^TM^ were confirmed by a Multisizer 3 device (Beckman Coulter, Brea, CA, USA) [23,24,25]. The morphology of DOX-bubbles was verified by a fluorescence microscope (IX-71, Olympus, Shinjuku-ku, Tokyo, Japan).

The signal-to-noise ratio (SNR) of DOX-bubbles was used to determine the in vitro acoustic stability acquired by an ultrasonic imaging system (7.5 MHz, model t3000, Terason, Middlesex, MA, USA). The DOX-bubbles were first loaded into a channel within a 2% agarose phantom. The channel of the phantom was constructed by embedding a dialysis tube (BD Corp., Franklin Lakes, NJ, USA; diameter: 1 mm) before phantom congealment. Once the agar gel had congealed, the tube was withdrawn, then leaving a wall-less tubular void. The images were then obtained periodically with a time interval of 10 min under 37 °C for 1 h. The SNR value was estimated from the contrast region within B-mode image.

When microbubble was destructed by US, it was referred to as inertial cavitation, and would emit acoustic broadband noise during microbubble collapse. Therefore, a 15-MHz US transducer (V303, Olympus, Waltham, NY, USA) was used to acquire the acoustic emission signals generated from microbubble under 1-MHz US exposure (model HS-3031, HES, Tainan, Taiwan; element size: 12 mm, peak-negative pressure: 0.1–1.0 MPa) for estimating the inertial cavitation threshold of microbubble. The focal zone of 1-MHz US was with a width of 9.7 mm and a length of 23 mm. The acquired signals were then translated to frequency spectra via fast Fourier transform with MATLAB (The Mathworks, Natick, MA, USA) to verified the occurrence of broadband noise. The resonance frequency of DOX-bubbles was evaluated according the previous method with peak-negative pressure of 0.1 MPa [26]. The leakage of DOX from DOX-bubbles was assessed through evaluating the fluorescent intensity of the DOX-bubble suspension at each time point using a plate reader system (Safire, Tecan, AG, Switzerland) at 596 nm. The drug retention with DOX-bubbles was estimated using the ratio of DOX leakage amount to the initial DOX loading amount.

The encapsulated DOX concentration of each DOX-bubble sample was calculated based on the fluorescence intensity of DOX. Before the measurement, the samples were sonicated to rupture the bubble structures and to avoid the densely encapsulated DOX molecules self-quenched in the lipid membranes.

The acoustic pressures used in this study were measured using a polyvinylidene difluoride type hydrophone (model HGL-0085, ONDA, Sunnyvale, CA, USA; calibration range: 1–40 MHz; spatial resolution: 85 µm) in an acrylic water tank that was filled with distilled and degassed water at 25 °C.

### 2.4. In vitro Anti-Tumor Effect of DOX-Bubbles and Setting up the US Sonication System

The MG-63 (CRL-1427) human osteosarcoma cell line was purchased from American Tissue Culture Collection (ATCC, Rockville, MD, USA) and cultured with Dulbecco’s modified Eagles medium (HG-DMEM) and 10% FBS. Prior to starting the experiment, 10^4^ of cells were seeded in a 96-well plastic plate (96-well Microtest^TM^ Plate, BD Falcon^TM^, Corning, NY, USA) and incubated with 5% CO_2_ in 37 °C.

**Figure 2:**
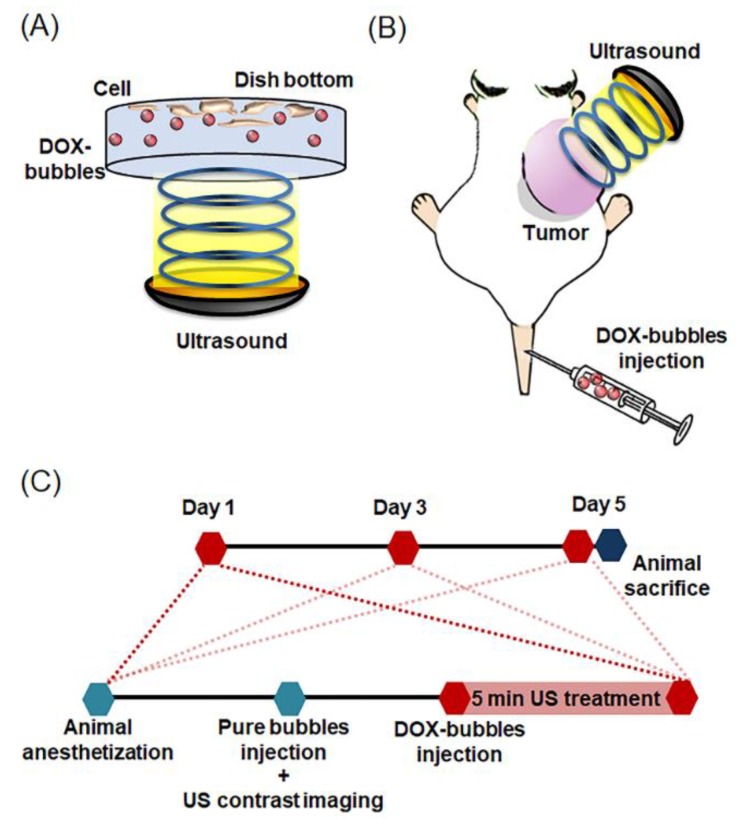
Experimental setup of (**A**) cell experiment and (**B**) animal tumor treatment. (**C**) Diagram of the osteosarcoma animal tumor treatment protocol.

Normal saline, DOX, and DOX-bubbles were co-cultured with the cells for 20 min followed by 1-MHz US sonication (peak-negative pressure: 0.3 MPa, pulse repetition frequency: 1 Hz, pulse length: 10 ms, duty cycle: 1%, time: 1 min). So as to prevent the formation of standing wave, a plate made by ultrasound absorption material was placed on the bottom of cell plate during the treatment. After a 20-min incubation period, the cells were washed and refilled with fresh media. The experimental setup is illustrated in Figure 2A. Cytotoxicity was estimated using Alamar Blue (AbDSerotec, Oxford, UK). For determining the transmitted waveform and the acoustic pressure of this system, a 1-MHz transducer was calibrated using a hydrophone (model HGL-0085, ONDA, Sunnyvale, CA, USA) at 25 °C.

### 2.5. In vivo Osteosarcoma Treatment

#### 2.5.1. In-situ Osteosarcoma Tumor Model

The immune-deficient NU-Foxn1nu mice used in our study protocols were purchased from the LASCO Laboratory (Taipei, Taiwan) and further cloned in specific pathogen-free condition at the Taipei Veterans General Hospital Animal Facility (Taipei, Taiwan) under the approved animal welfare and steps (IACUC 2012–188). Each mouse was IM injected with MG-63 cells through the right tibia from the knee joint for 1 × 10^7^ cells/0.1 mL PBS at 8 weeks of age [27]. Before starting experiment, mice were anesthetized by IP injecting 50 µL of Zoletil 50 (Virbac, Carros, France) /Rompun (Bayer HealthCare, Leverkusen, Germany) the mixed solution (50:50 vol%).

#### 2.5.2. Ultrasound Imaging and Evaluation of Tumor Microcirculation

To validate the capability of the DOX-bubbles in tumor microcirculation enhancement, a three-dimensional (3D) RSP6-16D transducer-equipped Voluson E8 ultrasound (GE healthcare, Ireland, UK) was used to obtain power Doppler volume histograms that were automatically calculated into indexes of vascularity (VI), flow (FI), and perfusion (PI). VI, represents the ratio of color-coded voxels to all voxels in the tumor. FI is the mean value of all color-coded voxels in the vessels of the volume analyzed. PI represents the mean value of voxels within the tumor region. The value of each color-coded voxel is expressed by the US instrument in arbitrary units (AU) on a scale of 0 to 100. These indexes were detected by DOX-bubbles and were further compared with values from pure bubbles and SonoVue^TM^ to comprehend the effect of bubble size on microcirculation enhancement. During the comparisons, each animal received DOX-bubbles, pure bubbles, and SonoVue^TM^ sequentially. The 3D Doppler scanning processes were performed at a mechanical index of 0.08 so as to avoid bubble destruction during the continuous scanning, and we also changed the injection order of these agents in each comparison to minimize the variance. The measurement was finished within 10 min.

#### 2.5.3. Osteosarcoma Treatment Procedures

Each treatment was initiated when the tumor volume reached 0.5 cm^3^. Three groups were evaluated including a control without treatment group (N-control, N = 6), DOX-bubble only group (DOX-bubbles only, N = 6), and a group that received DOX-bubbles with US insonation (DOX-bubbles + US, N = 6). The distance between the transducer surface and tumor was set at 15 mm. Before starting the treatment, each mouse underwent tumor microcirculation detection by pure bubbles (30 μL) with CEUS. Thirty minutes later, the mice were treated using DOX-bubbles (30 μL) with US. The time intervals between imaging and treatment allowed the bubbles to mostly clear from the circulation. The experimental setup and protocol are shown in Figure 2B,C. The tumor-bearing animal was placed prone, directly under a water cone with a 15 × 15 mm^2^ window on the bottom, sealed with a polyurethane membrane to allow the entry of US treatment pulse. The tumor was tightly attached to the membrane window. A US coupling gel was applied between the tumor and the membrane to maximize the transmission of US between the transducer and the brain. The treatment repeated 3 times at day 1, 3, and 5. The parameters of US treatment were set as previously described (Section 2.4). All insonations were set at 5 min and performed under the use of ultrasound gel to couple with the acoustic impedance.

#### 2.5.4. Histological Observation

Histological observation was utilized to monitor the morphology of the tumor tissues from different groups. The tumor tissues were harvested after finishing the imaging evaluation on day 5. Cryosection of the tissues was then performed at 20 μm thickness, and sections were mounted on glass slides. After the preparation, the slides were stained with hematoxylin and eosin (H&E stain, Sigma-Aldrich, St. Louis, MO, USA).

### 2.6. Statistics

All results are represented as the mean ± standard deviation. All statistical evaluations were carried out with unpaired two-tailed Student’s t-tests. A *p* value of less than 0.05 was referred to a significant difference.

## 3. Results

### 3.1. Characteristics of DOX-bubbles

**Figure 3:**
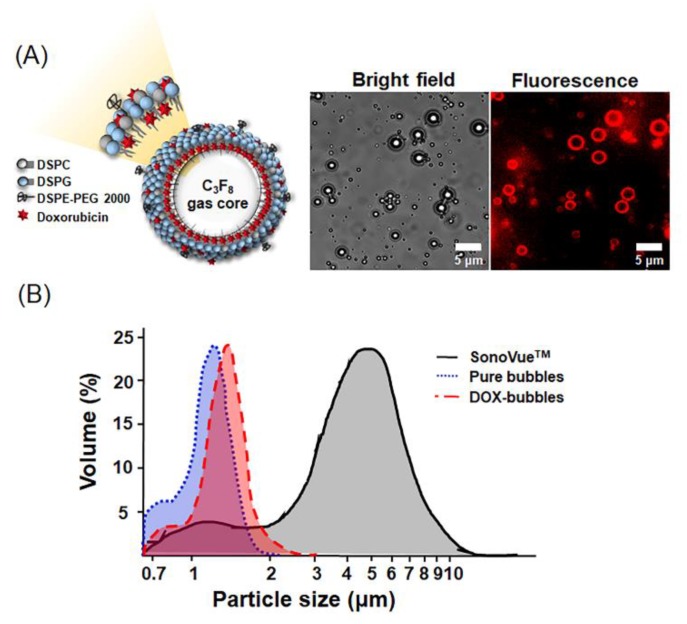
Size distribution and morphology of DOX (doxorubicin)-bubbles. (**A**) Left: the of DOX-bubbles; right: the bright field and fluorescent images of DOX-bubbles. Both of the images show the sphere shape of the DOX-bubbles, and the fluorescent image indicates the DOX molecules were incorporated in the lipid membranes. (**B**) Size distribution of pure bubbles, SonoVue^TM^, and DOX-bubbles.

The high co-localization of the bubbles, morphology and DOX fluorescence distribution suggests a successful combination of DOX and bubbles (Figure 3A). The mean size and concentration of the pure bubbles were 1.0 ± 0.2 μm and 33.2 ± 1.4 × 10^9^ bubbles/mL, respectively. The loading of DOX slightly enlarged the bubble size (1.1 ± 0.3 µm), and decreased the concentration (18.7 ± 5.9 × 10^9^ MBs/mL) (Figure 3B). For comparison, the mean size and concentration of SonoVue were 5.6 ± 0.9 μm and 6.6 ± 2.4 × 10^8^ bubbles/mL, respectively. The DOX loading efficiency was 69.4 ± 9.0%, and the final loaded DOX was around 1.38 mg/mL.

**Figure 4:**
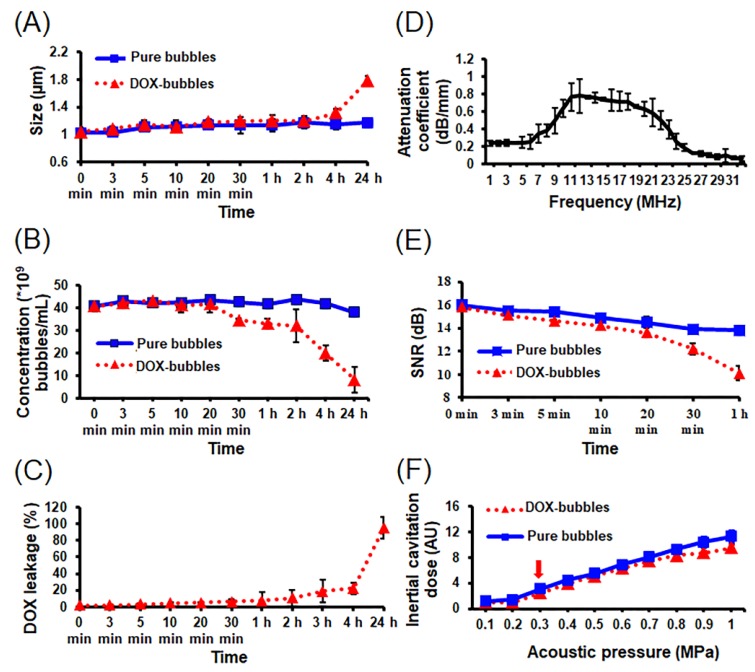
Properties of DOX-bubbles and pure bubbles. Size distribution (**A**) and concentration (**B**) of the two bubbles measured via Coulter counter at 37 °C at different time point. (**C**) Leakage of DOX from DOX-bubbles at 37 °C at different time points. (**D**) Attenuation measurements representing the resonance of DOX-bubbles with respect to the frequency of ultrasonic exposure. (**E**) In vitro acoustic stability of DOX-bubbles and pure bubbles. (**F**) The acoustic destruction threshold of DOX-bubbles and pure bubbles.

The pure bubbles demonstrated stability over a 24 h period (size: from 1.0 ± 0.1 to 1.2 ± 0.8 μm; concentration: from (41.4 ± 2.8) × 10^9^ bubbles/mL to (42.9 ± 1.3) × 10^9^ bubbles/mL) (Figure 4A). The size of DOX-bubbles showed stability at 4 h (from 1.1 ± 0.1 to 1.3 ± 0.1 μm) that significantly increased at 24 h (1.8 ± 0.1 μm). The concentration of DOX-bubbles started decreasing at 30 min from 90% ((34.6 ± 0.5) × 10^9^ bubbles/mL) to 20% ((8.2 ± 4.2) × 10^9^ bubbles/mL after 24 h (Figure 4B). DOX leakage started from 10.4 ± 9.8% at 2 h to 18.7 ± 0.6% at 3 h and 98.2 ± 12.5% after 24 h (Figure 4C). Figure 4D shows the resonance frequency of DOX-bubbles was about 11 -19 MHz. The acoustic stability of DOX-bubble remained relatively high until 20 min (0 min: 15.8 ± 0.1 dB; 30 min: 12.2 ± 0.5 dB; 1 h: 10.1 dB ± 0.6 dB). Since DOX delivery from DOX-bubbles needs the destruction of the DOX-bubbles by US, the US destruction threshold of DOX-bubbles was estimated. The inertial cavitation of DOX-bubbles was appeared when the acoustic pressure of US up to 0.3 MPa, indicating the onset of DOX-bubble collapse (Figure 4F). There were no differences in the destruction threshold between DOX-bubbles and pure bubbles. Therefore, we used 0.3 MPa of US sonication for the following experiments. These data also concluded that the properties of the bubbles were not affected by the encapsulation of DOX.

### 3.2. Controlled DOX Intracellular Delivery by DOX-Bubbles with US

**Figure 5:**
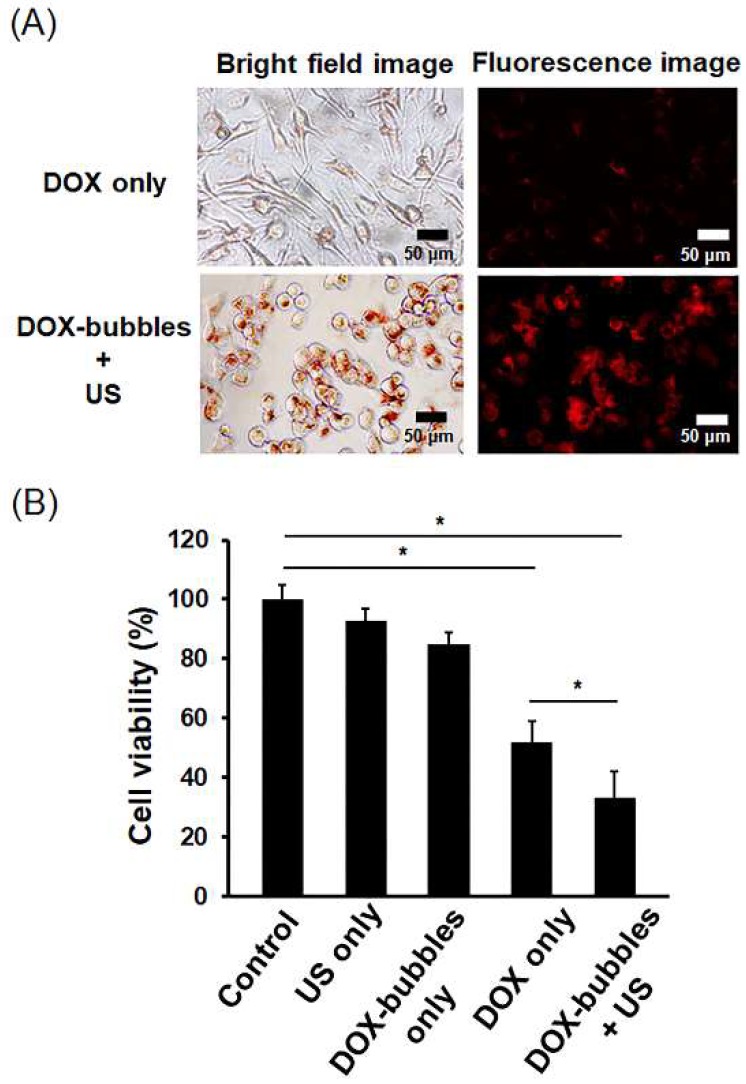
In vitro antitumor effect of DOX-bubbles and US (ultrasound) sonication. (**A**) Bright-field and fluorescent images of MG-63 cells treated with DOX, and DOX-bubbles + US. (**B**) Cell viability after treatment. Single asterisk, *p* < 0.05.

Next, the controlled drug release capability of DOX-bubbles upon US exposure was investigated in MG-63 cells. The fluorescent images confirmed the intracellular deposition of DOX in DOX-bubbles + US group, indicating that the encapsulated DOX could be triggered delivery into cells in conjunction with US exposure (Figure 5A). Cell viability was unaffected when US was applied alone. Administration with DOX alone caused a lower cell viability (52.5 ± 7.9%). DOX-bubble incubation alone produced a minor decrease in cell viability (85.5% ± 4.4%), likely because of the natural drug leakage from DOX-bubbles. The cytotoxicity of DOX could also be restricted by the protection of bubbles. Nevertheless, the combination between DOX-bubbles and US sonication also could provide anti-tumor ability (33.7 ± 9.9%) (Figure 5B), indicated that the DOX released from DOX-bubbles still had cytotoxicity. These data suggest that the DOX embedded in DOX-bubbles could be triggered for release by US so as to kill off the tumor cells.

### 3.3. In vivo Monitoring of Tumor Microcirculation Perfusion and Volume by DOX-Bubbles with CEUS

**Figure 6:**
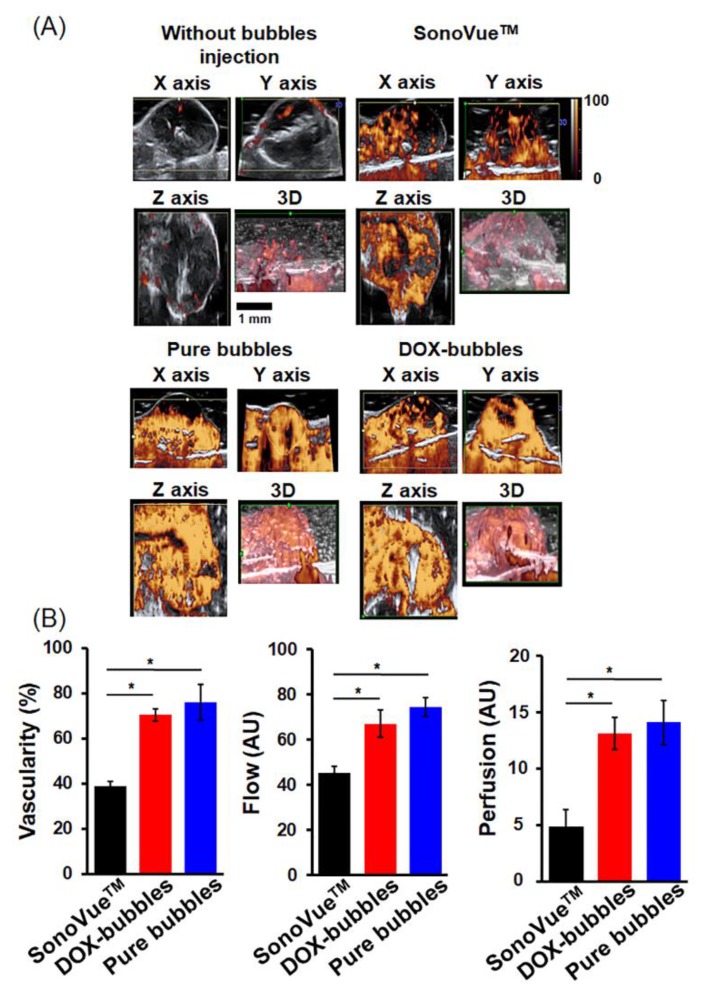
(**A**) The Doppler ultrasound images of osteosarcoma tumor model. Three groups were involved, without bubbles injection, SonoVue^TM^, pure bubbles, and DOX-bubbles. Note that the scale bar was for all the sub-figures. (**B**) Left: vascularity index (VI), denotes the ratio of color-coded voxels to all voxels of the tumor region; middle: flow index (FI), denotes the mean value of all color-coded voxels in the vessels of the volume analyzed; right: perfusion index (PI), denotes the mean value of voxels within the tumor region. These results suggested that compared with the SonoVue^TM^, DOX-bubbles showed better microcirculation enhancement. Single asterisk, *p* < 0.05.

Next, we evaluated the tumor microcirculation enhancement of DOX-bubbles with CEUS. The SonoVue^TM^ was used as the current standard for comprehending the effect of bubble size on microcirculation detection. Note that the concentrations of these bubbles were different (pure-bubbles: 33.2 ± 1.4 × 10^9^ bubbles/mL, DOX-bubbles: 18.7 ± 5.9 × 10^9^ bubbles/mL, SonoVue: 6.6 ± 2.4 × 10^8^ bubbles/mL), so we injected these bubbles with different volumes to ensure that the number of bubbles in each group was the same. The final injected volume of pure-bubbles, DOX-bubbles and SonoVue^TM^ were 20 μL, 35 μL and, 100 μL, respectively. Figure 6A shows the contrast signal intensity increment after administrating DOX-bubbles, and the same imaging technique could be further combined with 3D scanning to illustrate the volume information. These results suggest that compared with the SonoVue^TM^, both pure bubbles and DOX-bubbles showed a better microcirculation enhancement. The VI, FI, and PI of DOX-bubbles were 70.4 ± 2.8%, 67.1 ± 6.1 AU, and 13.1±2.9 AU, respectively (Figure 6B). The VI, FI, and PI of pure bubbles were 75.9 ± 8.8%, 76.9 ± 10.5 AU, and 14.4 ± 3.3 AU, respectively. For the current standard, SonoVue^TM^, the VI, FI, and PI were 39.8 ± 3.8%, 45.2 ± 3.3 AU, and 4.9 ± 1.5 AU, respectively. All the differences between these three indexes between DOX-bubbles and SonoVue^TM^ were statistically significant. There were no differences in these three indexes between DOX-bubbles and pure bubbles. This result indicates that the DOX-bubbles could be a reliable contrast media for tumor microcirculation enhancement and volume detection since it provided the missing information when a typical larger sized agent is used. Further, it might have the functionality to identify a small change in tumor vascularity before and after chemotherapies or the purposed concurrent OS theranostic strategy in this study.

### 3.4. In vivo OS Treatment Outcome and Concurrent Theranostic Strategy with DOX-Bubbles

**Figure 7:**
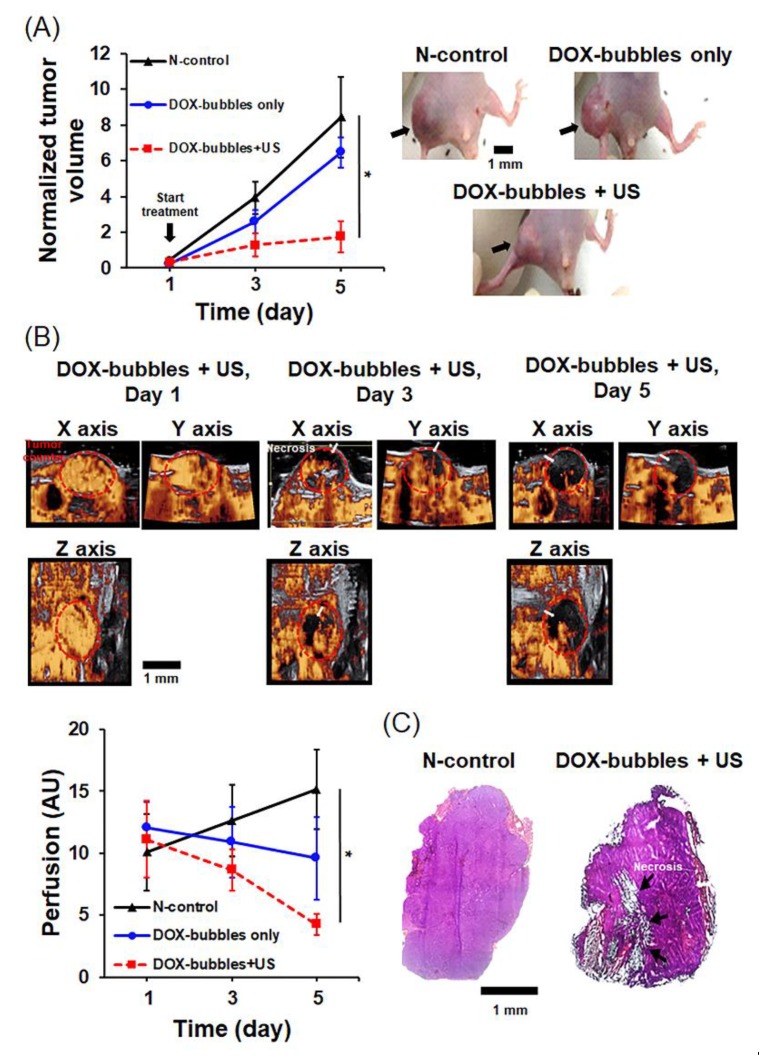
(**A**) Left: the therapeutic efficacy on the suppression of tumor volume; right: The macroscopic observation of the osteosarcoma tumor models of three groups. (**B**) The contrast-enhanced ultrasound imaging (CEUS) images (upper) and the tumor perfusion (bottom) before and after treatments with pure bubbles injection (the largest tumor section obtained from each scanning). White arrow: necrotic regions. Red dotted region: tumor contour. (**C**) Histological observation of the tumor after treatment. Black arrows: necrotic regions. Single asterisk, *p* < 0.05.

The therapeutic efficacy results are shown in Figure 7A. The normalized therapeutic efficacy is defined as the mean volume change between the experimental group and negative control. Only the group of DOX-bubbles + US could provide a significant suppression of tumor growth (day 1: 0.4 ± 0.1 cm^3^; day 3: 1.3 ± 0.7 cm^3^; day 5: 1.8 ± 0.9 cm^3^), and the normalized therapeutic efficacy was increased 3.7-fold compared with the group without treatment (N-control group, day 1: 0.5 ± 0.3 cm^3^, day 3: 3.9 ± 0.9 cm^3^, day 3: 8.5 ± 2.2 cm^3^ vs. DOX-bubbles only group, day 1: 0.4 ± 0.1 cm^3^, day 3: 2.6 ± 0.9 cm^3^, day 5: 6.5 ± 2.4 cm^3^). We also compared the overall perfusions of all groups between day 1 and day 5 to investigate the feasibility of using CEUS to evaluate the therapeutic outcome. Figure 7B demonstrates that the treatment of DOX-bubbles + US resulted in a significant decline of blood flow in the tumor area, suggesting the occurrence of DOX-induced necrosis. We also noticed an interesting trend, that only the perfusion of N-control group increased within 5 days from 10.1 ± 3.8 AU to 15.2 ± 4.9 AU. The overall perfusions of both groups that received DOX-bubbles were reduced from 12.1 ± 2.1 to 9.6 ± 3.4 AU for the group of DOX-bubble only group, and 11.3 ± 3.2 to 4.3 ± 1.9 AU for the group of DOX-bubbles + US. The macroscopic and histological observations shown in Figure 7C also agreed with the imaging data. These outcomes provide evidence that the synergistic effect of DOX-bubbles with US could contribute to suppression of tumor growth and acceleration of tumor necrosis. Furthermore, the therapeutic outcome could be monitored by pure bubbles with CEUS, suggesting the theranostic application of our proposed DOX-bubbles.

## 4. Discussion

### 4.1. Significance

We showed the efficacious use of DOX-loaded bubbles for transporting DOX in combination with US sonication as a theranostic approach for OS treatment. The fabricated DOX-bubbles showed better tumor microcirculation enhancement than commercial bubble SonoVue^TM^ with CEUS imaging. Additionally, the tumor growth was suppressed by treatment with DOX-bubbles and US in only five days because of the acceleration of the DOX entering to tumor cells and leading necrosis process. This study provides a new non-invasive approach for delivering chemotherapeutic drug in OS treatment.

### 4.2. Preparation of DOX-Bubbles

The chemotherapeutic drug (DOX) loading onto the lipid shells of bubbles relies on electrostatic and hydrophobic interactions. The red fluorescence of DOX-bubbles revealed successful complexation of the cationic microbubbles with DOX (Figure 3). Our data conclude that the stability and acoustic property of bubbles were not affected by the encapsulation of DOX. In addition, the cytotoxicity of DOX could be reduced after the encapsulation into bubbles, regardless of US irradiation. On the other hand, the size of bubbles did not obviously change before and after DOX loading (pure bubbles: 1.0 ± 0.2 µm vs. DOX-bubbles: 1.1 ± 0.3 µm). It was reported that the microbubbles might be aggregated forming large clusters or coalesce into larger bubbles during long-pulsed US stimulation [28,29,30]. These effects might induce gas embolism within circulation, blocking local blood flow and decreasing drug delivery efficiency. Future works should verify the occurrence of these effects during treatment and adjust the US treatment parameters to control the clustering process. The small size of the DOX-bubbles produced better tumor microcirculation enhancement as well as potentially increased the safety during treatment. Previous reports have indicated that the bio-effects from bubbles could be reduced by decreasing the size of the bubbles because larger bubbles can easily expand and fragment while in contact with the endothelial cells [31,32].

### 4.3. US Treatment

Previous studies have demonstrated that ultrasound exposure can trigger drug release from drug-loaded bubbles and also enhance drug permeability of tumor vessels through the cavitation effect [33,34]. This effect might be the most important difference between free drugs and the bubble-based drug delivery systems. The disruption of bubbles caused both inertial and stable cavitation to induce micro-streaming and liquid jets. These mechanical forces stimulate shrinkage of vessels and increased drug permeability [35,36,37]. To avoid the heating effect from high-intensity and continuous insonation, we used low-intensity (MI = 0.3) and pulsed (pulse repetition frequency = 1 Hz) US insonation parameters to perform the treatment. Thus, we believe that the therapeutic outcome in the experimental group of DOX-bubbles + US was driven by chemotherapeutics and stimulation. Note that pulsed ultrasound is used to avoid the heating effect of ultrasound and provide a sufficient time interval for the reperfusion of DOX-bubbles [38,39]. Through a by-side ultrasound imaging system, we monitored the reperfusion of the DOX-bubbles for estimating the treatment outcome. However, the results also indicated that ultrasound insonations are needed since only the group of DOX-bubbles + US significantly suppressed tumor growth. Therefore, physicians can probably use the therapeutic DOX-bubbles to simultaneously perform both perfusion evaluation and treatment in a real clinical setting. Thus, DOX-bubbles can be used as a theranostic agent for concurrent osteosarcoma theranosis. Conversely, previous studies had reported the lifetime of microbubbles and the spatiotemporal uniformity of cavitation activity by short-pulse ultrasound [40,41], suggesting that the distribution of cavitation perhaps could be adjusted by the waveform of US. Therefore, the future work includes resulting in efficient therapies by spreading cavitation throughout the treatment area with code-excitation US pulse.

### 4.4. Limitations

The limitations of the purposed strategy might lie on the penetration depth of ultrasound. To demonstrate the attenuation of 1-MHz ultrasound, we measured the attenuated acoustic intensity during penetration of a 5-mm-thick pork humerus, and the result showed that the acoustic pressure would be reduced to 0.1 MPa (refers to 7.13 dB, data not shown). In contrast to 1-MHz ultrasound, we also tested a 3-MHz transducer using the same setups, and the acoustic pressure was dramatically reduced from 1.1 MPa to 0.1 MPa (refers to 20.83 dB, data not shown). The inertial cavitation threshold of bubbles is typically around 0.1 to 0.2 MPa when using low frequency ultrasound (less than 3 MHz). This comparison reveals that lower frequencies (lower than 1 MHz) or higher intensity (higher than 1 MPa) might be more useful for osteosarcoma treatments. The new technology, high-intensity focused ultrasound (HIFU), which is now currently used to ablate the bone tumors [42,43,44], has become more popular in recent years. The local acoustic pressure of HIFU could be heightened to more than 10 MPa for direct penetration into tumor tissues. Thus, the combination of DOX-bubbles and HIFU might be another way to perform concurrent osteosarcoma theranosis. However, planar ultrasound is probably more suitable for regional treatments than for larger insonation areas. As a result, a planar ultrasound device that can transmit high-intensity ultrasound is critical for launching a concurrent osteosarcoma theranostic strategy in a clinical setting. Another limitation of the current study was the absence of flow in the in vitro experiments. The blood flow would affect the microbubble dynamics and the pressure thresholds required to achieve intracellular delivery [45]. Besides, the in vitro experiments were conducted by monolayer cell structure, making it was hard to refer to tumor conditions. Future works include designing an in vitro flow system or tumor-chip device for observing the potential mechanisms of US + drug-loaded bubbles regulated drug delivery under flow condition.

## 5. Conclusions

In this study, we established an approach using drug-loaded bubbles for concurrent osteosarcoma theranosis. The combination of low-intensity (0.3 MPa) US and drug-loaded bubbles provides an efficient controlled release strategy for providing 3.7-fold suppression of tumor growth compared with the group without US exposure (1.8 ± 0.9 cm^3^ vs. 8.5 ± 2.2 cm^3^). Using CEUS technology, we also found that the proposed drug-loaded bubbles showed 1.8-fold of vascularity (70.4 ± 2.8 % vs. 39.8 ± 3.8 %), 1.7-fold of flow (67.1 ± 6.1 AU vs. 45.2 ± 3.3 AU), and 2.7-fold of perfusion (13.1 ± 2.9 AU vs. 4.9 ± 1.5 AU) higher than commercialized microbubbles in tumor microcirculation detection. This study provided a novel theranostic strategy for US regulated tumor drug delivery into clinical use.
